# Revealing geographical and population heterogeneity in HIV incidence, undiagnosed HIV prevalence and time to diagnosis to improve prevention and care: estimates for France

**DOI:** 10.1002/jia2.25100

**Published:** 2018-03-30

**Authors:** Lise Marty, Françoise Cazein, Henri Panjo, Josiane Pillonel, Dominique Costagliola, Virginie Supervie, the HERMETIC Study Group, Hanne Apers, Jessika Deblonde, Anda Ķīvīte, Jasna Loos, Christiana Nöstlinger, Daniela Rojas Castro, Dominique Van Beckhoven

**Affiliations:** ^1^ INSERM Sorbonne Université Institut Pierre Louis d'Epidémiologie et de Santé Publique Paris France; ^2^ Santé publique France French National Public Health Agency Saint‐Maurice France; ^3^ Paris Sud University Orsay France; ^4^ Centre de Recherche en Epidémiologie et Santé des populations (CESP) INSERM U1018 Villejuif France

**Keywords:** HIV incidence, undiagnosed HIV prevalence, time to HIV diagnosis, subnational and sub‐population estimates, modelling, exposure group, geographical analysis, map

## Abstract

**Introduction:**

To close gaps in HIV prevention and care, knowledge about locations and populations most affected by HIV is essential. Here, we provide subnational and sub‐population estimates of three key HIV epidemiological indicators, which have been unavailable for most settings.

**Methods:**

We used surveillance data on newly diagnosed HIV cases from 2004 to 2014 and back‐calculation modelling to estimate in France, at national and subnational levels, by exposure group and country of birth: the numbers of new HIV infections, the times to diagnosis, the numbers of undiagnosed HIV infections. The denominators used for rate calculations at national and subnational levels were based on population size (aged 18 to 64) estimates produced by the French National Institute of Statistics and Economic Studies and the latest national surveys on sexual behaviour and drug use.

**Results:**

We estimated that, in 2014, national HIV incidence was 0.17‰ (95% confidence intervals (CI): 0.16 to 0.18) or 6607 (95% CI: 6057 to 7196) adults, undiagnosed HIV prevalence was 0.64‰ (95% CI: 0.57 to 0.70) or 24,197 (95% CI: 22,296 to 25,944) adults and median time to diagnosis over the 2011 to 2014 period was 3.3 years (interquartile range: 1.2 to 5.7). Three mainland regions, including the Paris region, out of the 27 French regions accounted for 56% of the total number of new and undiagnosed infections. Incidence and undiagnosed prevalence rates were 2‐ to 10‐fold higher than the national rates in three overseas regions and in the Paris region (*p*‐values < 0.001). Rates of incidence and undiagnosed prevalence were higher than the national rates for the following populations (*p*‐values < 0.001): born‐abroad men who have sex with men (MSM) (respectively, 108‐ and 78‐fold), French‐born MSM (62‐ and 44‐fold), born‐abroad persons who inject drugs (14‐ and 18‐fold), sub‐Saharan African‐born heterosexuals (women 15‐ and 15‐fold, men 11‐ and 13‐fold). Importantly, affected populations varied from one region to another, and in regions apparently less impacted by HIV, some populations could be as impacted as those living in most impacted regions.

**Conclusions:**

In France, some regions and populations have been most impacted by HIV. Subnational and sub‐population estimates of key indicators are not only essential to adapt, design implement and evaluate tailored HIV interventions in France, but also elsewhere where similar heterogeneity is likely to exist.

## 1 Introduction

Over the past two decades, remarkable progress has been achieved in the fight against HIV. The life expectancy of people living with HIV has dramatically increased since effective combination antiretroviral treatment (cART) has been available 1 and continues to improve 2. Additionally, effective cART, by reducing the HIV viral load in body fluids to undetectable levels, prevents onward HIV transmission 3. Other new prevention tools, such as Pre‐Exposure Prophylaxis (PrEP), that is the use of antiretroviral drugs by HIV‐negative individuals, have also shown high effectiveness 4, 5. These advances in HIV treatment and prevention have the potential to dramatically reduce the number of new HIV infections and could open a new path towards the end of AIDS epidemic. This prompted the Joint UN Program on HIV/AIDS (UNAIDS) to launch the ambitious 90‐90‐90 target 6, calling for 90% of people living with HIV to know their HIV status, 90% of them to be on cART and 90% of them to have achieved viral suppression. However, to reach this target by 2020, and eventually end the AIDS epidemic, it will require ensuring rapid and early access to cART as well as extending tailored prevention services.

In most settings affected by HIV, including high‐income countries, late cART initiation remains common 7, 8, 9, 10, 11, 12. Current major obstacles to early access to cART are undiagnosed and late‐diagnosed HIV infections 11, 12, 13. Undiagnosed and late‐diagnosed individuals are at risk of HIV/AIDS‐related morbidity and mortality 14, 15, and transmitting HIV 16, 17 because of delayed cART initiation. Ensuring early access to cART will hence require shrinking the time interval from infection to HIV diagnosis, by raising awareness about the issue of undiagnosed and late‐diagnosed HIV infections among the civil society and implementing relevant testing programmes, to increase uptake of HIV testing among people who may have undiagnosed HIV and/or be at risk of being diagnosed late. This necessitates having a comprehensive understanding of the populations affected by undiagnosed and late‐diagnosed HIV infections as well as whether these populations change from one location to another. Likewise, extending prevention services requires identifying the populations at risk of HIV acquisition in each geographic area, and providing them with prevention tools tailored to their needs 18.

Some efforts have been undertaken to describe the HIV epidemics at a finer scale. Most studies focused on the geographic analysis and mapping of the HIV prevalence 19, 20, 21, 22 and HIV diagnoses 23, 24. Similar efforts to produce subnational and sub‐population estimates of HIV incidence, time from infection to diagnosis and the undiagnosed population, which are the key epidemiological indicators to guide and evaluate HIV prevention and testing programmes, remained to be investigated in most settings; only the US Centers for Disease Control and Prevention has recently generated estimates for HIV incidence and undiagnosed prevalence at the state‐level 22. More importantly, determining, within a country, whether and how the populations affected by HIV differ from one geographic area to another remained to be done.

In this study, we provide a geographical and sub‐population analysis of three key epidemiological indicators: HIV incidence, distribution of times from HIV infection to diagnosis and prevalence of undiagnosed HIV infections, and a description of the populations affected by HIV according to the geographical area. To conduct this analysis, we used national surveillance data on newly diagnosed HIV cases in France 23 and a, previously developed, statistical model 25, 26.

## 2 Methods

### 2.1 Data source

In France, like in many high‐income countries, routine HIV surveillance is based on case reporting of all new HIV diagnoses, and conducted by Santé publique France, the French national public health agency. It was implemented in March 2003 27. Data on new HIV diagnoses include date of diagnosis, demographic information (sex and country of birth), HIV exposure group, clinical status at diagnosis (primary HIV infection (PHI), asymptomatic, symptomatic without AIDS or AIDS) and region of residence. Reported cases are adjusted for delay in reporting, under‐reporting and missing data 28.

### 2.2 HIV incidence and distribution of time from infection to HIV diagnosis

To estimate HIV incidence and the distribution of time from infection diagnosis, we used a back‐calculation model and data on newly diagnosed HIV cases. The model has been described elsewhere 25 and is detailed in Appendix S1 and S2. Note that in our approach we estimated the distribution of time from infection diagnosis for individuals who were newly infected in a specific year (and not for individuals who were diagnosed in a specific year), and throughout the article, we used the term distribution of time from infection diagnosis to refer to this distribution. Briefly, our model relies on the principle that trends in newly diagnosed HIV cases reflect both trends in HIV incidence and the distribution of times from infection to diagnosis. Thus, the former can be used to estimate the latter. However, due to the lack of identifiability in distinguishing changes in HIV incidence from changes in distribution of times from infection to diagnosis, some estimates may be unreliable, unless extra information on one of the two indicators to estimate (HIV incidence or the distribution of times from infection diagnosis) is incorporated into the model. In our approach, we used data on the clinical status at diagnosis to bring information on the distribution of time from infection to diagnosis. We considered three clinical status: PHI, AIDS, neither PHI nor AIDS. Individuals diagnosed with PHI were assumed to have short time intervals from infection to diagnosis (3 months in median) while individuals diagnosed with AIDS were assumed to have longer time intervals, and depending on the natural AIDS incubation time (10 years in median). Individuals diagnosed without PHI or AIDS were assigned with intermediate, but unknown, time intervals, depending on two parameters representing uptake of routine testing and onset of HIV symptoms that occur towards the end of the incubation period. Values for these two parameters together with HIV incidence were then simultaneously estimated by fitting the back‐calculation model to the observed numbers of new HIV diagnoses stratified by clinical stage.

### 2.3 Number of undiagnosed HIV infections

To estimate the number of individuals living with undiagnosed HIV, we used estimates of HIV incidence and distribution of times from infection to diagnosis. The method has been described elsewhere 26 and is detailed in Appendix S3 in SM. Briefly, the estimated number of new HIV infections was projected forward according to the distribution of times from infection to diagnosis to obtain estimates of the number of individuals living with undiagnosed HIV infection in a given year. Specifically, from the number of newly HIV‐infected individuals at each point in time, we estimated those who were still undiagnosed in a given year, using the cumulative probabilities of not being diagnosed with HIV over time; these probabilities were calculated from the distributions of time between infection and diagnosis.

### 2.4 Producing estimates at the national and subnational level and by population

We first used national data on newly diagnosed HIV cases from 2004 to 2014 (Figure S1A) to obtain estimates of the three epidemiological indicators at the national level. Then, we used data on newly diagnosed HIV cases from 2004 to 2014 of each region of France to produce regional estimates; in 2014, there were 27 regions in France, including 21 mainland regions, Corsica and five overseas regions: Guyane in South America (also known as French Guyana), Guadeloupe and Martinique in the Caribbean, and Reunion and Mayotte in the Indian Ocean. We also produced, at the national level and in four selected regions, estimates by sex, HIV exposure group and country of birth. The exposure group is determined based on what individuals declare as the probable mode of HIV acquisition when diagnosed with HIV. If more than one mode is reported, the case is classified in the exposure category listed first in the following hierarchy: man who have sex with men (MSM), person who injects drugs (PWID) and heterosexual. For the country of birth, we split individuals between those born in France and those born abroad, and among those born broad we also produced estimates for individuals born in Sub‐Saharan Africa (SSA). The four regions, namely Guyane, Ile‐de‐France (the Paris region), Provence‐Alpes‐Côte d'Azur and Centre, were selected to illustrate contrasted situations regarding the burden of HIV epidemic and the populations most affected by HIV (Figure S1B). We produced mean and 95% confidence intervals (CI) using a bootstrap procedure (see Appendix S4 in SM). Note that in the results section, we present our estimates for the year 2014 for incidence and undiagnosed HIV prevalence, while for the time to diagnosis, we chose to present the distribution for individuals infected between 2011 and 2014, and not only in 2014, because the time to diagnosis can greatly vary from year to year, but also variation may not be pertinent to inform testing strategies.

The denominators used for rate calculations at the national and subnational level were based on population size (aged 18 to 64) estimates in 2014 produced by the French National Institute of Statistics and Economic Studies 29, which also provides estimates according to the country of birth, and estimates from the latest national surveys on sexual behaviour 30 and drug use 31. Note that for the denominator of the incidence rate we subtracted the HIV prevalence 12 from the population size. We defined MSM as men who had at least one sexual intercourse with another man in the past 12 months and PWID as people who injected drugs in the past 12 months. More details are given in SM on population size estimates, calculation of rates and statistical comparisons (see Appendix S5 to S7).

## 3 Results

In 2014, in France, an estimated 6607 new infections occurred, corresponding to an incidence rate of 1.7 per 10,000 (Table 1). There were an estimated 24,197 individuals living with undiagnosed HIV, corresponding to an undiagnosed prevalence rate of 6.1 per 10,000 (Table 1). The median time from infection to diagnosis for individuals infected from 2011 to 2014 was 3.3 years (interquartile range (IQR): 1.2 to 5.7).

**Table 1:** National‐level estimates of HIV incidence and undiagnosed HIV prevalence in 2014 and time to diagnosis for individuals infected between 2011 and 2014 for France, by sex, HIV exposure group and country of birth

	Number of new HIV infections (95% CI)	Median time (in years) from infection to diagnosis (IQR)	Number of undiagnosed HIV infections (95% CI)	Population size^a^ (18 to 64 year) (95% CI)	Incidence rate per 10,000 (95% CI)	Prevalence of undiagnosed HIV per 10,000 (95% CI)
MSM (all)	2938 (2651 to 3290)	2.7 (0.5 to 5.0)	9181 (8065 to 10,125)	311,480 (248,800 to 382,887)	115.1 (89.6 to 141.9)	298.5 (233.9 to 379.7)
MSM born in France	2302 (2041 to 2628)	2.6 (0.5 to 5.0)	7157 (6111 to 8044)	268,770 (214,684 to 330,385)	104.5 (80.3 to 128.4)	269.6 (210.0 to 343.2)
Born‐abroad MSM^b^	636 (491 to 817)	3.2 (1.2 to 5.5)	2025 (1624 to 2459)	42,710 (34,115 to 52,501)	181.8 (130.7 to 243.4)	480.2 (352.8 to 662.7)
Born‐abroad heterosexual women (all)	1399 (1200 to 1626)	3.1 (1.3 to 5.1)	5159 (4398 to 5786)	2,861,832 (2,859,872 to 2,863,092)	5.0 (4.3 to 5.6)	18.0 (15.4 to 20.2)
Heterosexual women born in SSA	1029 (844 to 1209)	2.9 (1.2 to 4.9)	3811 (3270 to 4354)	416,700 (416,414 to 416,883)	25.1 (20.6 to 28.8)	91.5 (78.5 to 104.4)
Born‐abroad heterosexual men (all)	1221 (933 to 1604)	4.5 (2.4 to 6.8)	5265 (4409 to 6446)	2,607,948 (2,597,354 to 2,617,203)	4.7 (3.6 to 5.9)	20.2 (16.9 to 24.7)
Heterosexual men born in SSA	871 (657 to 1113)	4.2 (2.1 to 6.4)	3565 (2943 to 4316)	459,234 (457,369 to 460,864)	19.2 (14.5 to 23.7)	77.6 (63.9 to 94.0)
Heterosexual women born in France	403 (291 to 523)	3.0 (1.0 to 5.4)	1526 (1254 to 1828)	17,111,885 (17,100,164 to 17,119,422)	0.2 (0.2 to 0.3)	0.9 (0.7 to 1.1)
Heterosexual men born in France	561 (323 to 825)	4.6 (1.9 to 7.0)	2711 (2108 to 3476)	16,411,594 (16,344,929 to 16,469,836)	0.3 (0.2 to 0.5)	1.6 (1.3 to 2.1)
PWID (all)	86 (27 to 211)	4.0 (1.8 to 6.5)	357 (217 to 657)	104,317 (80,727 to 132,800)	10.2 (3.0 to 21.9)	34.8 (19.5 to 63.2)
PWID born in France	59 (10 to 169)	4.3 (2.1 to 6.7)	205 (91 to 512)	89,860 (69,541 to 114,382)	8.0 (1.2 to 18.2)	23.1 (9.5 to 56.4)
PWID born abroad^b^	28 (5 to 69)	3.5 (1.5 to 6.0)	153 (79 to 270)	14,458 (11,185 to 18,418)	23.6 (4.5 to 51.4)	107.7 (55.0 to 202.6)
Total men	4776 (4273 to 5335)	3.4 (1.1 to 5.8)	17,394 (15,723 to 19,147)	19,410,383	2.5 (2.2 to 2.7)	9.0 (8.1 to 9.9)
Total women	1831 (1606 to 2123)	3.1 (1.2 to 5.0)	6803 (6026 to 7546)	19,998,673	0.9 (0.8 to 1.0)	3.4 (3.0 to 3.8)
Total	6607 (6057 to 7196)	3.3 (1.2 to 5.7)	24,197 (22,296 to 25,944)	39,409,056	1.7 (1.5 to 1.8)	6.1 (5.7 to 6.6)

CI, confidence interval; IQR, interquartile range; MSM, men who have sex with men; PWID, persons who inject drugs; SSA, Sub‐Saharan Africa.

a Details are given in Appendix S5 in SM on how population sizes were obtained.

b For born‐abroad MSM and PWID, we did not produce estimates of incidence, time to diagnosis and undiagnosed prevalence according to the country of birth; however, it is interesting to note that among newly diagnosed HIV cases, over 2009 to 2014, 28% of born‐abroad MSM were born in Haiti or in the Americas, 26% in Europe, 26% in North Africa or Asia or Oceania, and 19% in SSA, and 72% of born‐abroad PWID were born in Europe.

### 3.1 Geographical heterogeneity

**Figure 1:**
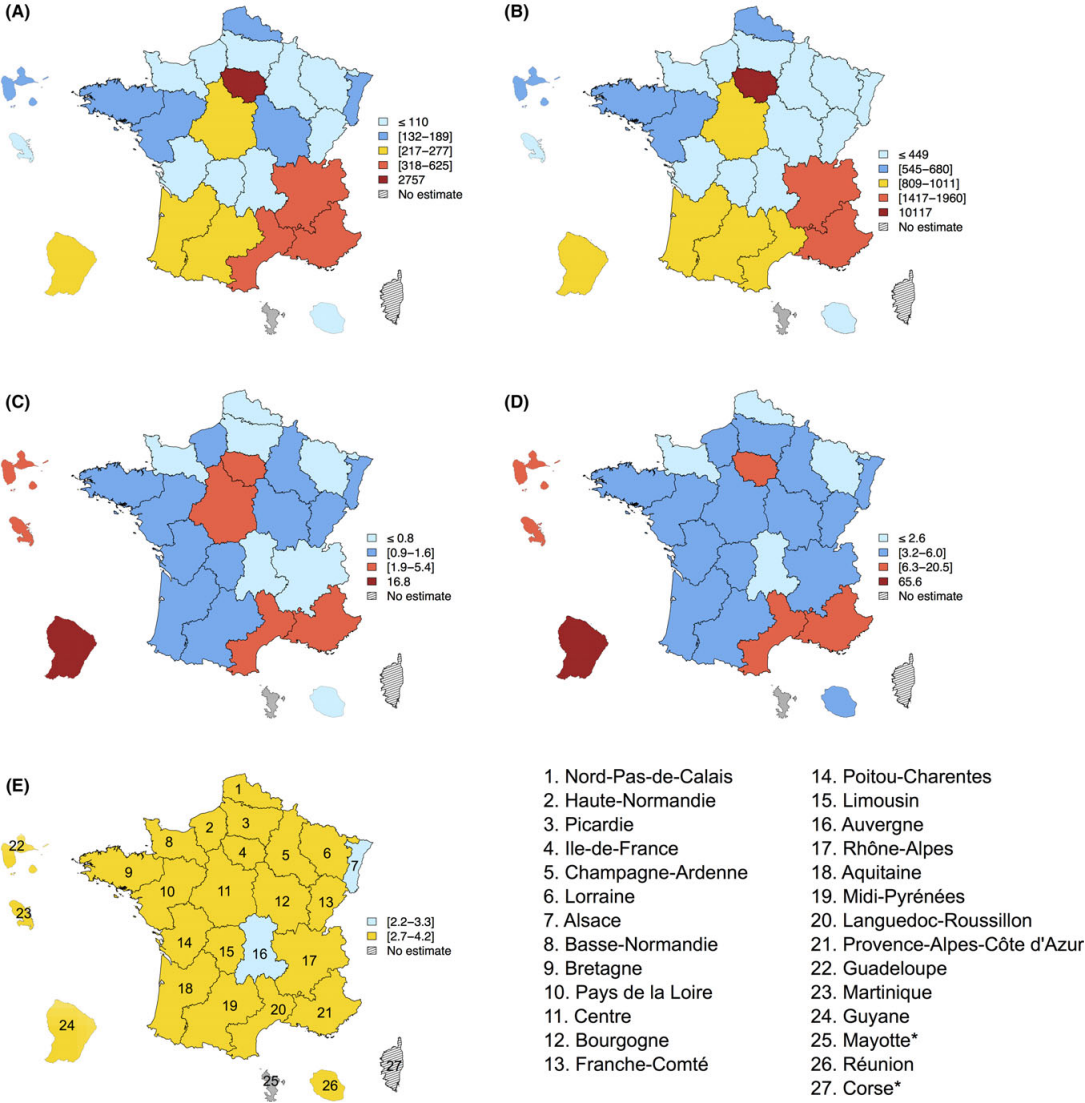
Subnational estimates for France. (A) Number of new HIV infections in 2014; (B) number of undiagnosed HIV infections in 2014; (C) number of new HIV infections per 10,000 inhabitants (aged 18 to 64 years old) in 2014; (D) number of undiagnosed HIV infections per 10,000 inhabitants (aged 18 to 64 years old) in 2014; (E) median time between infection and diagnosis (in years) for individuals infected between 2011 and 2014. *We could not provide estimates for Corse and Mayotte because of incomplete surveillance data. In (A) and (B), yellow‐coloured regions correspond to regions with numbers close to average number of new infections individuals in A (i.e. 6607/27≈245) and undiagnosed HIV individuals in B (i.e. 24,197/27 ≈ 896), and in (E) to regions with distributions of times from infection to diagnosis not statistically different than the national‐level distribution; Estimated national rates were 1.7 per 10,000 (95% CI: 1.5 to 1.8) for HIV incidence, 6.1 per 10,000 (95% CI: 5.7 to 6.6) for undiagnosed HIV infections and the national‐level median time between infection and diagnosis was 3.3 years (interquartile range: 1.2 to 5.7). In (A–D), light blue (respectively dark blue) coloured regions correspond to regions with numbers or rates more than twice lower (respectively less than twice lower) than average numbers or national rates, while dark red (respectively light red) coloured regions correspond to regions with numbers or rates more than nine times higher (respectively less than nine times higher) than average numbers or national rates. In (E), light blue‐coloured regions correspond to regions with distribution of times from infection to diagnosis statistically different than the national‐level distribution, with values for the median lower than or equal to that of the national level. The maps were produced using the package maptools in R 3.2.4 39.

Forty‐two percent of the estimated new infections (2757 out of 6607) occurred among people living in Ile‐de‐France (Figure 1A).

Two regions of the south‐east of France, Provence‐Alpes‐Côte d'Azur and Rhône‐Alpes, accounted for 14% of the estimated new infections (respectively 625 and 318 new infections). It is in these three regions that were living 56% of the undiagnosed individuals (Figure 1B): Ile‐de‐France region (42%, 10,117 out of 24,197), Provence‐Alpes‐Côte d'Azur (8%) and Rhône‐Alpes (6%). Relating these numbers to the size of the population living in each region, we found that seven regions had at least one rate higher than national level (*p*‐values < 0.0001, red‐coloured regions on Figure 1C and D). Guyane had rates (per 10,000 inhabitants) of incidence and undiagnosed prevalence around ten times higher (respectively 16.8 and 65.6), followed by Guadeloupe with rates three times higher (respectively 5.4 and 20.5), Ile‐de‐France and Martinique with rates around two times higher (respectively 3.7 and 13.4 for Ile‐de‐France, 4.3 and 14.0 for Martinique), and Provence‐Alpes‐Côte‐d'Azur (respectively 2.2 and 6.8) and Languedoc‐Roussillon (respectively 2.0 and 6.3) with rates significantly higher than the national level (*p*‐value < 0.0001). Centre had slightly but significantly higher incidence rate than the national level (1.9; *p*‐value < 0.0001, red‐coloured region on Figure 1C). Time from infection to diagnosis for individuals infected from 2011 to 2014 remained long and not significantly different than national level in most regions (yellow‐coloured regions on Figure 1E), and significantly different in two regions (blue‐coloured regions on Figure 1E) with median lower or equal than the national level in Alsace (2.2 years) and Auvergne (3.3 years), (*p*‐values < 0.0001).

### 3.2 Population heterogeneity

At the national level, while MSM, heterosexuals and PWID represented, respectively, 0.8%, 98.9% and 0.3% of the general population, in 2014, 44% of the new HIV infections (2938 out of 6607) and 38% of undiagnosed infections (9181 out of 24,197) occurred among MSM, respectively, 54% and 61% among heterosexuals, and 1% and 1% among PWID, respectively (Table 1). Among MSM, 22% of the new infections or undiagnosed infections occurred among born‐abroad MSM. Among PWID, about 33% of the new infections and 43% undiagnosed infections occurred among born‐abroad PWID. Regarding born‐abroad heterosexuals, among women more than 70% of the new infections or undiagnosed infections occurred among SSA‐born women and among men around 70% of the new infections or undiagnosed infections occurred among SSA‐born men. When considering rates (per 10,000) of incidence and undiagnosed prevalence, the most affected populations were born‐abroad MSM (with respective rates of 181.8 and 480.2), MSM born in France (respectively 104.5 and 269.6), born‐abroad PWID (respectively 23.6 and 107.7) and SSA‐born heterosexual women (respectively 25.1 and 91.5) and men (respectively 19.2 and 77.6). The rates of HIV incidence and undiagnosed HIV prevalence were higher than the national rates for each of these populations (*p*‐values < 0.0001): 108‐ and 78‐fold, 62‐ and 44‐fold, 14‐ and 18‐fold, 15‐ and 15‐fold, 11‐ and 13‐fold respectively. Among individuals infected between 2011 and 2014, heterosexual men and PWID born in France had longer time to diagnosis than MSM and heterosexual women (medians of >4 years versus ≤3.2 years) but it was only statistically significant for heterosexual men (*p*‐values < 0.0001 for all two‐by‐two comparisons).

### 3.3 Geographical and population heterogeneity

To illustrate existing heterogeneity in how populations are affected by HIV according to the geographic area, we chose to focus on four selected regions.

In Ile‐de‐France, rates of incidence and undiagnosed prevalence were, respectively, 195.9 and 450.7 for born‐abroad MSM, 151.2 and 344.7 for MSM born in France, 28.6 and 98.2 for SSA‐born heterosexual women, 19.6 and 88.7 for SSA‐born heterosexual men, and 17.9 and 54.4 for PWID (Table 2). Heterosexual men and PWID had longer time from HIV infection to diagnosis than MSM and heterosexual women (medians of ≥4 years *vs*. ≤3 years) but it was only statistically significant for heterosexual men (*p*‐values < 0.05 for two‐by‐two comparisons except heterosexual men born in France against heterosexual women born in France).

**Table 2:** Subnational‐level estimates of HIV incidence and undiagnosed HIV prevalence in 2014 and time to diagnosis for individuals infected between 2011 and 2014 by sex, HIV exposure group and country of birth for four selected French regions

	Ile‐de‐France				Provence‐Alpes‐Côte d'Azur				Centre				Guyane			
	Pop. size^a^ (18 to 64 year)	IR (95% CI)	UP (95% CI)	Median TI (IQR) in years	Pop. size^a^ (18 to 64 year)	IR (95% CI)	UP (95% CI)	Median TI (IQR) in years	Pop. size^a^ (18 to 64 year)	IR (95% CI)	UP (95% CI)	Median TI (IQR) in years	Pop. size^a^ (18 to 64 year)	IR (95% CI)	UP (95% CI)	Median TI (IQR) in years
MSM (all)	93,178 (61,341 to 134,306)	163.3 (104.1 to 226.0)	373.4 (237.4 to 557.9)	2.4 (0.5 to 4.7)	43,515 (24,506 to 70,525)	94.0 (49.7 to 145.0)	213.9 (114.5 to 384.3)	2.0 (0.4 to 4.3)	7011 (3211 to 13,195)	244.6 (98.0 to 447.1)	600.1 (264.8 to 1132.9)	2.8 (0.8 to 5.3)	xx	X	X	X
MSM born in France	67,989 (44,759 to 98,000)	151.2 (91.0 to 208.2)	344.7 (211.1 to 547.2)	2.2 (0.4 to 4.5)	35,554 (20,023 to 57,623)	102.1 (53.2 to 154.4)	223.9 (118.3 to 386.5)	1.9 (0.4 to 4.2)	6304 (2887 to 11,863)	X	X	X	xx	X	X	X
Born‐abroad MSM	25,188 (16,582 to 36,306)	195.9^b^ (120.7 to 285.3)	450.7^b^ (275.2 to 696.1)	2.8^b^(1.0 to 5.0)	7961 (4483 to 12,902)	57.8^b^ (7.3 to 116.2)	169.4^b^ (58.8 to 367.1)	2.5^b^ (0.7 to 4.8)	707 (324 to 1331)	X	X	X	xx	X	X	X
Born‐abroad heterosexual women (all)	1,080,951 (1,080,543 to 1,081,327)	6.5 (5.3 to 7.7)	22.7 (19.4 to 26.1)	2.9 (1.2 to 4.9)	280,313 (279,971 to 280,571)	3.4 (1.3 to 6.2)	9.1 (5.0 to 17.6)	3.0 (1.2 to 5.1)	78,027 (77,985 to 78,054)	4.9 (1.6 to 9.4)	23.4 (13.4 to 36.2)	3.3 (1.6 to 5.1)	32,794	23.9^c^ (11.7 to 36.6)	94.8^c^ (65.5 to 132.1)	3.0^c^ (1.4 to 5.1)
Heterosexual women born in SSA	212,146 (212,066 to 212,220)	28.6 (22.7 to 33.5)	98.2 (81.9 to 113.8)	2.8 (1.2 to 4.8)	26,169 (26,137 to 26,193)	X	X	X	13,143 (13,136 to 13,147)	27.0 (7.0 to 54.9)	128.8 (71.9 to 215.1)	3.3 (1.6 to 5.1)	770	X	X	X
Born‐abroad heterosexual men (all)	963,350 (951,696 to 972,082)	5.5 (3.7 to 7.3)	26.3 (21.6 to 32.9)	4.4 (2.3 to 6.6)	248,176 (242,816 to 251,824)	5.8 (1.7 to 10.3)	16.5 (7.9 to 33.1)	4.5 (2.3 to 6.8)	73,644 (73,006 to 74,053)	X	X	X	30,573	26.0^c^ (8.4 to 47.7)	109.4^c^ (62.6 to 176.5)	4.1^c^ (2.0 to 6.5)
Heterosexual men born in SSA	227,655 (224,901 to 229,719)	19.6 (12.7 to 25.9)	88.7 (71.3 to 108.4)	4.2 (2.1 to 6.3)	29,654 (29,013 to 30,090)	X	X	X	15,118 (14,987 to 15,202)	X	X	X	635	X	X	X
Heterosexual women born in France	2,795,141 (2,794,084 to 2,796,114)	0.5 (0.3 to 0.7)	1.7 (1.2 to 2.3)	3.0 (1.0 to 5.4)	1,207,109 (1,205,637 to 1,208,222)	0.7 (0.3 to 1.4)	1.5 (0.6 to 3.6)	2.6 (0.9 to 4.5)	674,966 (674,599 to 675,200)	X	X	X	38,787	X	X	X
Heterosexual men born in France	2,600,331 (2,568,873 to 2,623,902)	0.6 (0.3 to 1.0)	2.7 (1.8 to 4.0)	4.1 (1.4 to 6.6)	1,108,378 (1,084,438 to 1,124,669)	0.6 (0.2 to 1.4)	2.7 (1.5 to 5.4)	4.2 (1.8 to 6.7)	656,254 (650,563 to 659,892)	X	X	X	38,447	X	X	X
PWID^c^	25,742 (20,723 to 30,602)	17.9 (1.9 to 45.6)	54.4 (21.0 to 131.5)	4.0 (1.9 to 6.5)	15,343 (10,229 to 21,242)	X	X	X	2382 (1410 to 3581)	X	X	X	xx	X	X	X
Total men	3,676,384	5.3 (4.4 to 6.0)	19.5 (16.4 to 22.7)	3.5 (1.2 to 5.8)	1,411,695	3.3 (2.5 to 4.2)	11.1 (8.8 to 13.8)	3.0 (0.8 to 5.5)	738,717	3.1 (2.0 to 4.1)	8.9 (6.4 to 12.5)	3.2 (1.1 to 5.9)	69,020	19.6 (9.3 to 31.7)	78.7 (51.1 to 116.3)	4.3 (2.1 to 6.6)
Total women	3,882,308	2.1 (1.7 to 2.5)	7.6 (6.4 to 8.9)	3.0 (1.3 to 5.1)	1,491,138	1.1 (0.6 to 1.6)	2.7 (1.7 to 3.9)	2.7 (1.0 to 4.9)	753,568	0.7 (0.2 to 1.2)	3.1 (2.0 to 4.6)	3.1 (1.2 to 5.1)	71,581	14.1 (8.1 to 20.1)	57.0 (42.4 to 76.6)	3.2 (1.5 to 5.3)
Total	7,558,692	3.7 (3.2 to 4.1)	13.4 (11.8 to 15.1)	3.3 (1.2 to 5.6)	2,902,833	2.2 (1.7 to 2.7)	6.8 (5.5 to 8.2)	2.9 (0.8 to 5.4)	1,492,285	1.9 (1.2 to 2.5)	5.9 (4.5 to 7.9)	3.2 (1.1 to 5.7)	1,406,001	16.8 (10.1 to 24.0)	65.6 (49.9 to 84.2)	3.7 (1.8 to 6.0)

MSM, men who have sex with men; PWID, persons who inject drugs; SSA, Sub‐Saharan Africa; CI, confidence interval; IQR, interquartile range; IR, HIV incidence rates (per 10,000 inhabitants aged 18 to 64); UP, undiagnosed HIV prevalence rates (per 10,000 inhabitants aged 18 to 64); TI, time interval from HIV infection to diagnosis for individuals infected between 2011 and 2014; X, values could not be estimated due to insufficient HIV cases; xx, the 2006 French national survey on sexual behaviour 30 and the 2014 survey on drug use 31 did not provide estimates of the sizes of the MSM and PWID populations in overseas regions.

a Details on how population sizes were obtained and confidence intervals for the population size are given in Appendix S5 in SM.

b For born‐abroad MSM, we did not produce estimates of incidence, time to diagnosis and undiagnosed prevalence according to the country of birth; however, it is interesting to note that among newly diagnosed HIV cases, in Ile‐de‐France, 29% of born‐abroad MSM were born in Haiti or in the Americas, 29% in North Africa or Asia or Oceania, 21% in Europe, and 20% in SSA, and in Provence‐Alpes‐Côte d'Azur 41% were born in Europe and 28% in North Africa or Asia or Oceania. ^#^For PWID, estimate could not be produced according to the country of birth due to insufficient HIV cases.

c In Guyane, for born‐abroad heterosexuals, we did not produce estimated of incidence, time to diagnosis and undiagnosed prevalence according to the country of birth; however, among heterosexuals, most born‐abroad newly diagnosed HIV cases were born in Haïti or in the Americas (84%).

In Provence‐Alpes‐Côte d'Azur, born‐abroad MSM and MSM born in France had by far the highest rates (per 10,000) of incidence (respectively 57.8 and 102.1) and undiagnosed prevalence (respectively 169.4 and 223.9) compared to other groups (*p*‐values < 0.0001, Table 2). Heterosexual men tended to have longer times from HIV infection to diagnosis than other groups (medians of >4 years *vs*. ≤3 years for the other groups, *p*‐values < 0.05 for all two‐by‐two comparisons for heterosexual men born in France against MSM born in France and born‐abroad heterosexual men against born‐abroad heterosexual women).

In the region Centre, we could only produce estimates by sex, and for two groups, MSM and born‐abroad heterosexual women that represented, respectively, 37% and 27% of the new HIV diagnoses (Figure S1B). Rates (per 10,000) of incidence and undiagnosed prevalence were, respectively, 244.6 and 600.1 for MSM and 27.0 and 128.8 for SSA‐born heterosexual women (Table 2).

In Guyane, we could only produce estimates by sex, and for two groups, born‐abroad heterosexual women and men that represented, respectively, 40% and 30% of the new HIV diagnoses (Figure S1B). Rates (per 10,000) of incidence and undiagnosed prevalence were, respectively, 23.9 and 94.8 for born‐abroad heterosexual women and 26.0 and 109.4 for born‐abroad heterosexual men (Table 2). Time from infection to diagnosis tended to be longer among born‐abroad heterosexual men than born‐abroad heterosexual women (median of 4.1 years *vs*. 3.0, *p*‐value = 0.4636).

## 4 Discussion

In France, substantial heterogeneity exists in HIV incidence, undiagnosed HIV prevalence and time from infection to diagnosis across locations and populations. These features of the French HIV epidemics need to be accounted for to improve prevention and care in France. To the best of our knowledge, this is the first study quantifying within‐country geographical and population heterogeneity for these three key epidemiological indicators.

HIV affects all regions of France, but four regions (three overseas and one mainland) were more affected than others. By far, the most impacted region was Guyane, located in South America, with HIV incidence and undiagnosed HIV prevalence rates around 10 times higher than national rates. Next, it was Guadeloupe, an island in the Caribbean, followed by Martinique, another island in the Caribbean, and Ile‐de‐France, which includes Paris, the biggest city of France, with rates two to three times higher than national level. Remarkably, more than 40% of the new and undiagnosed HIV infections occurred among people living in Ile‐de‐France, while only 19% of the French population (aged 18 to 64) lives in this region. HIV affects all populations but five populations were more affected than others. By far, the most affected populations were born‐abroad MSM and MSM born in France, with undiagnosed HIV prevalence and HIV incidence rates 44‐ to 108‐fold higher than the national level. Then, it was born‐abroad persons who inject drugs and SSA‐born heterosexuals (men and women), with rates 11‐ to 18‐fold higher than the national level.

The populations most affected by HIV differed from one region to another and in regions that were apparently less affected than the four aforementioned, some populations were as impacted by HIV as those living in the most impacted area. In Ile‐de‐France, the most impacted populations were MSM, whether born in France or abroad, followed by SSA‐born heterosexual women and men, and PWID. In Provence‐Alpes‐Côte d'Azur, it was mainly MSM, whether born in France or abroad, while in Centre it was both MSM and SSA‐born heterosexual women. In these last two regions, incidence and undiagnosed HIV prevalence rates for MSM and SSA‐born heterosexual women were as high as those estimated for these populations in Ile‐de‐France. In Guyane, the most impacted populations were heterosexual men and women born in Haiti or in the Americas. A common feature for all regions was that times from infection to diagnosis for individuals remained long for all populations, and especially for heterosexual men, whether born in France or abroad, who had, or tended to have, longer time intervals from infection to diagnosis.

The main strength of our approach is that it only requires data on newly diagnosed HIV cases. Our approach can be applied to any country collecting data on the annual number of newly diagnosed HIV cases as well as the clinical status at diagnosis, exposure group and country of birth, which includes most high‐income countries 32. Several things were done to check the internal and external validity of our results (see Appendix S8 of the SM), nevertheless, our results are subject to a number of limitations affecting surveillance data and data used to determine the size of populations. As surveillance data on new HIV cases were not exhaustive, they were adjusted for missing entries, reporting delay and under‐reporting 28. Potential inaccurate adjustment could affect our estimates. Furthermore, as French surveillance system on newly diagnosed HIV cases was paper‐based until recently, it was labour intensive and time‐consuming to collect data, and thus, it generated gaps in data availability, which prevented producing more timely estimates. Our findings reflect then the epidemiological situation in 2014, and thus attitudes towards HIV testing and prevention until that year. If these attitudes have changed since 2014, for example with use of PrEP, which was made available in France in 2016, it could have altered estimates for HIV infection, and to a lower extent estimates for the undiagnosed prevalence. One could also argue that universal treatment, which was implemented in France at the end of 2013, had influenced behaviours towards HIV testing, yet in the past, no major changes in HIV testing behaviours were observed when treatment eligibility guidelines were changed 33. Gap in data availability is not an issue specific to France, but rather a global issue. Addressing this issue, and improving the timeliness of surveillance data availability for analysis and dissemination, for instance developing a national‐based electronic surveillance system—this was done in France in 2016—should be a priority, because real‐time epidemiological estimates can be a powerful tool to design, monitor and evaluate interventions aiming to prevent HIV infection and/or reduce time to diagnosis.

In addition, estimates of the size of some populations, such as MSM and PWID, remain uncertain because they are imprecise at the subnational level and rely on definitions that may be too restrictive. We defined MSM and PWID based on sexual activity or drug use over the last twelve months. More inclusive definitions, based on sexual activity or drug use over the last two or five years, would have led to higher population sizes and lower rates. Moreover, national surveys 30, 31 provided estimates of the prevalence of MSM and PWID at the national and subnational level, but not according to the country of birth. We then assumed that the prevalence of MSM and PWID was similar among individuals born in France and those born abroad, but it remains unclear whether and how this assumption could have biased our results. Moreover, to produce regional estimates, we used the region of residence at diagnosis. While, it seems reasonable to assume that the region of residence at diagnosis brings some information on where individuals were living before being diagnosed, thus on where individuals were living with undiagnosed HIV infection, the region of residence at diagnosis does not bring any information on where individuals acquired HIV. Some individuals, especially among those born abroad, may have acquired HIV infections outside France. A recent study conducted among SSA migrants living in Ile‐de‐France showed that from 51% to 65% of them acquired HIV before migrating in France 34. Thus, we probably overestimated the number of new HIV infections that occurred in France, and thus HIV incidence rates, especially among born‐abroad individuals, and in regions, where the proportion of born‐abroad individuals among new HIV cases is high. Finally, our back‐calculation model accounts for some, and maybe not all, changes in test‐seeking behaviours that occurred over time. These changes are essentially driven by the time‐varying proportions of individuals diagnosed with PHI (see Appendix S2 in SM). By assuming that, for individuals diagnosed without PHI or AIDS, uptakes of HIV testing due to routine medical examination or onset of HIV symptoms that occur towards the end of the incubation period were constant over the 2004 to 2014 period, we may have failed to appreciate all the changes in test‐seeking behaviours. However, annual number of HIV tests performed in France remained fairly stable over the 2004 to 2014 period 33, suggesting that no major changes in test‐seeking behaviours occurred over this period.

Our results show that in France, a high number of new and undiagnosed HIV infections (>40%) are concentrated in a delimited geographical area, namely the region Ile‐de‐France. This offers the opportunity to intensify prevention and testing programmes in this area to eventually impact HIV transmission in this area, and possibly elsewhere in France. Indeed, recent phylogenetic studies concluded that the region Ile‐de‐France was the major hub of dissemination in France 35, 36, and that among clusters of primary HIV infections involving patients living in not contiguous regions 70% involved one patient living in Paris area 37. This suggests that reducing HIV transmission in Ile‐de‐France could indirectly reduce HIV transmission in other regions of France. Our findings also revealed that, for all HIV exposure groups (i.e. MSM, heterosexuals and PWID), born‐abroad individuals account for a significant fraction of the number of new and undiagnosed HIV infections. Moreover, when considering rates, born‐abroad populations are among the most affected by HIV. This calls for tailored HIV prevention and testing interventions towards these individuals and shows the importance of producing sub‐population estimates. Moreover, we found that in geographical areas that appear less impacted by HIV, some populations (e.g. born‐abroad MSM, SSA‐born women) can be as impacted by HIV than those living in the most impacted area, thus our findings also clearly invite to implement and target HIV prevention and testing programmes towards specific populations in each region of France, based on key epidemiological indicators, such as HIV incidence, undiagnosed HIV prevalence and time from infection to diagnosis. Finally, we found that times from infection to diagnosis remained long in all regions and for all populations, and especially for heterosexual men. Time to diagnosis could be an interesting surrogate metric to monitor access to cART in France, and probably in many other settings. Indeed, we previously showed that, in France, HIV diagnosis remained the main gap in the continuum of HIV care and thus the duration between HIV infection and cART initiation was highly dependent on the time to HIV diagnostic 12. As a consequence, monitoring the time to diagnosis and implementing interventions aiming at reducing this time will be essential to accelerate cART initiation, on which depends the success of treatment as prevention.

Similar geographical and population heterogeneity are likely to occur in other settings and need to be investigated. Unravelling these heterogeneity is key to impact HIV transmission at the population level, by intensifying intervention programmes in most affected geographical areas, and reduce individual‐level risk of HIV acquisition and time from infection to diagnosis, by targeting affected populations in each geographical area. Without a granular view of the HIV epidemics within countries, it will not be possible to close gaps in HIV prevention and care continuums, and thus end the HIV and AIDS epidemics. In this study, we used surveillance data on newly diagnosed HIV cases, which are mainly available in most high‐income countries. In other settings, there also exists surveillance systems collecting data on uptake of antiretroviral therapy, currently underused, for which back‐calculation models can also be developed to derive subnational estimates 38.

## 5 Conclusions

Our study clearly shows that the HIV epidemic in France is diverse. Risks of HIV acquisition, of undiagnosed HIV infections and time to HIV diagnosis are not uniformly distributed across geographical areas and populations. This highlights not only the need to design, adapt and/or implement subnational tailored HIV prevention and testing programmes based on key epidemiological indicators, such as HIV incidence, undiagnosed HIV prevalence and time from infection to diagnosis, in France, but also in other settings where similar heterogeneity is likely to exist.

## Competing interest

VS has served on advisory boards for ViiV Healthcare (2016) and reports lecture fees from Gilead (2014, 2015) and MSD (2014). DC reports grants from Janssen‐Cilag (2014), Merck‐Sharp & Dohme‐Chibret (2017), ViiV (2015), personal fees from Janssen‐Cilag (2016), Merck‐Sharp & Dohme‐Chibret (2015) for lectures, personal fees from Gilead (2014), ViiV (2015), Janssen‐Cilag (2014) for travel, accommodations and meeting expenses, personal fees from Gilead France from 2011 until December 2015 for French HIV board, personal fees from Innavirvax (2015 and 2016) for consultancy, outside the submitted work. LM, FC, HP and JP declare no conflicts of interest.

## Authors’ contributions

VS designed the research; LM performed the research; all authors analysed the data; LM, DC and VS drafted the manuscript; all authors critically revised the manuscript for important intellectual content.

## Supporting information


**Appendix S1.** Description of the back‐calculation model.
**Appendix S2**. Estimating the number of new HIV infections and the distribution of times from infection to diagnosis.
**Appendix S3**. Estimating the number of undiagnosed HIV‐infected individuals.
**Appendix S4.** Precision of the estimates and estimates of the HIV incidence before 2003.
**Appendix S5.** Estimating population sizes.
**Appendix S6.** Estimating prevalence rates of undiagnosed HIV and rates of HIV incidence.
**Appendix S7.** Statistical tests and maps.
**Appendix S8.** Internal and external validity.
**Table S1.** Population size, aged 18 to 64, by sex and origin, in 2014 in France at the national level and in four selected regions 5, 8
**Table S2.** Estimated proportion of men, aged 18 to 69, who had sex with another man in the past twelve months in France and in four selected regions in 2006 6
**Table S3.** Estimated number of individuals, aged 15 to 64, who injected drugs in the past twelve months, by sex, in France and in four selected regions in 2014 7
**Table S4.** Estimated population sizes and 95% confidence intervals, aged 18 to 64, by sex, HIV exposure group and origin, in France and in four selected regions
**Table S5.** Estimated national‐level rates of HIV prevalence in France in 2010 by sex, HIV exposure group and origin
**Table S6.** Annual expected and observed numbers of HIV diagnosis at the national level over 2004 to 2014
**Table S7.** Estimated time from infection to HIV diagnosis and CD4 cell count at HIV diagnosis by group
**Figure S1.** Surveillance data on newly diagnosed HIV cases. (A) Annual national number of newly diagnosed HIV cases and 95% confidence intervals from 2004 to 2014; (B) average annual number of new HIV diagnoses over the years 2009 to 2014 in each French region and percentage of new HIV diagnoses by HIV exposure group and origin. Overall, 10% of newly diagnosed HIV cases were diagnosed during the primary HIV infection (PHI), 15% with AIDS and 75% without AIDS or PHI.
