# Supplementary material for: Revealing geographical and population heterogeneity in HIV incidence, undiagnosed HIV prevalence and time to diagnosis to improve prevention and care: estimates for France
Source: J Int AIDS Soc. 2018 Mar 30;21(3):e25100. doi: 10.1002/jia2.25100 (PMC5878416; doi:10.1002/jia2.25100)

# Supplementary Material

**Revealing Geographical and Population Heterogeneity in HIV Incidence, Undiagnosed HIV Prevalence and Time to Diagnosis to Improve Prevention and Care:**

**Estimates for France**

Lise Marty^1^, Françoise Cazein^2^, Henri Panjo^3^, Josiane Pillonel^2^, Dominique Costagliola^1^, Virginie Supervie^1^ and the HERMETIC Study Group**

^1^INSERM, Sorbonne Université, Institut Pierre Louis d’Epidémiologie et de Santé Publique, 56 bd Vincent Auriol, F75013, Paris, France

^2^Santé publique France, French national public health agency, 12 Rue Val d'osne, F 94415, Saint-Maurice, France

^3^Paris Sud University, 15 Rue Georges Clemenceau, Orsay 91400, France; Centre de Recherche en Epidémiologie et Santé des populations (CESP), INSERM U1018, 16, avenue Paul Vaillant-Couturier, Villejuif 94807, France.

**HERMETIC Study Group: Hanne Apers (ITM, Belgium), Jessika Deblonde (WIV-ISP, Belgium), Anda Ķīvīte (RSU, Latvia), Jasna Loos (ITM, Belgium), Lise Marty (INSERM U1136, France), Christiana Nöstlinger (ITM, Belgium), Daniela Rojas Castro (AIDES, France), Virginie Supervie (INSERM U1136, France), Dominique Van Beckhoven (WIV-ISP, Belgium).

We describe in the sections S1 to S4, in a general way, our back-calculation model [[1](#_ENREF_1)] and how we used it to estimate the number of new HIV infection, the distribution of times from infection to HIV diagnosis, and the number of undiagnosed HIV infections [[2](#_ENREF_1)]. Note that in our approach we estimated the distribution of time from infection diagnosis for individuals who were newly infected in a specific year (and not for individuals who were diagnosed in a specific year), and throughout the text we used the term distribution of time from infection diagnosis to refer to this distribution. We used our approach to produce estimates for each group at the national and subnational level (e.g. men who have sex with men born in France and living in the region Centre, heterosexual women born abroad and living in Guyane, etc). The back-calculation model was written in C++ language using Xcode 6.3.2.

## S1. Description of the back-calculation model

### Assigning specific test-seeking behaviors to newly diagnosed individuals according to their clinical status at diagnosis.

We partitioned our quarterly data on newly diagnosed individuals according to the clinical status at diagnosis. We created two groups.

Group 1 consisted of individuals diagnosed with primary HIV infection (PHI). By definition, individuals belonging to group 1 were diagnosed very early in the course of the infection. We assumed that these individuals decided to be tested because they experienced, and/or recognized, symptoms of PHI, or because they had recently been exposed to HIV.

Group 2 consisted of individuals diagnosed without primary HIV infection (PHI). Among them, some individuals were diagnosed with AIDS and others without AIDS. We assumed that individuals diagnosed with AIDS were not found HIV positive before being diagnosed with AIDS while individuals who were diagnosed without AIDS decided to be tested for HIV for other reasons than those of individuals belonging to group 1 (e.g. routine medical examination or onset of symptoms that occur towards the end of the incubation period). It is important to note that in group 2, among individuals diagnosed without AIDS, there are HIV-infected individuals who were diagnosed during PHI but not identified as such, because they did not experience and/or report symptoms of PHI. In our approach, we then consider three kinds of clinical status at diagnosis: 1) PHI; 2) AIDS and 3) neither AIDS nor PHI.

### Specifying group-specific distributions of times from infection to diagnosis.

The next step consists in linking the observed number of newly diagnosed cases to the unobserved number of new HIV infections by specifying the distribution of times from infection to diagnosis for each of the two groups.

As for notation, $Y_{t,D}$ denotes the observed number of individuals diagnosed in quarter (3-months period) *t* with clinical status of type D, and $N_{s,D}$ denotes the unobserved number of individuals newly infected in quarter *s* that are diagnosed with clinical status of type D, i.e. PHI (P), AIDS (A), or neither AIDS nor PHI (H).

The unobserved number of individuals newly infected in quarter *s* is:

$N_{s}=N_{s,P}+N_{s,H}+N_{s,A}$ (1)

We assume that $N_{s}$ are realizations of independent Poisson variables with mean $\lambda_{s}$.

*Group 1*

For individuals diagnosed with PHI (group 1, G_1_), the duration $T_{H}$ from infection to diagnosis was assumed to be uniform from 0 to 6 months (i.e. the median length of the primary stage of HIV infection was assumed to be 3 months). Thus:

$E\left( Y_{t,P} \right)=\sum_{s=1}^{t} E(N_{s,P})\Pr(T_{P}=t-s)=\sum_{s=1}^{t} \lambda_{s}^{G_{1}} f_{t-s,P}$ (2)

where $\lambda_{s}^{G_{1}}=E(N_{s,P})$ is the mean number of new HIV infections in quarter *s* that are then diagnosed during the primary stage of HIV infection, $E(Y_{t,P})$ is the mean number of individuals diagnosed during the primary stage of HIV infection in quarter *t* and $f_{x,P}$ is the probability that an infected individual is diagnosed during the primary stage of HIV infection, *x* quarters after contracting HIV.

*Group 2*

For individuals diagnosed with AIDS and individuals diagnosed without AIDS or PHI (group 2) we adopted the same approach as Becker *et al.* [[3](#_ENREF_1)]. Basically, each (unobserved) newly HIV-infected individual belonging to group 2 was allocated, independently, a duration $T_{A}$ from infection to an AIDS diagnosis and a duration $T_{H}$ from infection to a diagnosis of HIV infection. Thus, a newly HIV-infected individual belonging to group 2 could either have AIDS when diagnosed, meaning that he/she was found HIV positive before being diagnosed with AIDS, or be diagnosed without AIDS, meaning that he/she did not develop AIDS before being tested. Assuming that each individual in group 2, infected during a quarter *s*, is independently assigned a time from HIV infection to diagnosis, leads to:

$E\left( Y_{t,H} \right)=\sum_{s=1}^{t} E\left( N_{s,A}+N_{s,H} \right)\Pr\left( T_{H}=t-s,T_{A}>t-s \right)=\sum_{s=1}^{t} \lambda_{s}^{G_{2}}f_{t-s,H}$ (3)

$E\left( Y_{t,A} \right)=\sum_{s=1}^{t} E\left( N_{s,A}+N_{s,H} \right)\Pr\left( T_{A}=t-s,T_{H}\geq t-s \right)=\sum_{s=1}^{t} \lambda_{s}^{G_{2}}f_{t-s,A}$ (4)

where $\lambda_{s}^{G_{2}}=E\left( N_{s,A})+E(N_{s,H} \right)$ is the mean number of new HIV infections in quarter *s* that are then diagnosed with AIDS, or without AIDS or PHI,$E(Y_{t,H})$ is the mean number of individuals diagnosed without AIDS or PHI in quarter *t*, $E(Y_{t,A})$ is the mean number of individuals diagnosed with AIDS in quarter *t*, $f_{x,H}$ is the probability that an HIV-infected individual is diagnosed without AIDS or PHI, *x* quarters after contracting HIV, without first developing AIDS, and $f_{x,A}$is the probability that an infected individual is diagnosed with AIDS, *x* quarters after contracting HIV, and was not tested for HIV before developing AIDS.

The time from infection to AIDS diagnosis, $T_{A}$, was assumed to follow a Weibull distribution with a median of 40 quarters (i.e. 10 years) [[4](#_ENREF_1)]:

$F_{A}\left( t \right)=1-exp[{-(0.0215t)}^{2.516}]$ (5)

The distribution of the rate of pre-AIDS HIV testing was assumed to depend on two unknown parameters that represent uptake of routine testing ($\nu)$ and onset of symptoms that occur towards the end of the incubation period $(\gamma)$:

$F_{H}\left( t \right)=1-exp[{-\nu t-\gamma(0.0215t)}^{2.516}]$ (6)

It is important to realize from equation (6) that we did not constraint to zero the probability of HIV testing within months after HIV infection for individuals supposedly diagnosed without AIDS or PHI. In consequence, our approach accommodates the situation where some HIV-infected individuals diagnosed during the primary stage of HIV infection do not report and/or show symptoms of PHI and thus are classified as not having PHI.

Assuming independence between the discrete random variables $T_{A}$ and $T_{H}$, the probabilities in equations (3) and (4), were then specified by:

$f_{t,H}=Pr\left( T_{H}=t,T_{A}>t \right)=\left[ F_{H}\left( t+0.5 \right)-F_{H}\left( t-0.5 \right) \right]{[1-F}_{A}\left( t \right)]$ (7)

$f_{t,A}=Pr\left( T_{A}=t,T_{H}\geq t \right)=\left[ F_{A}\left( t+0.5 \right)-F_{A}\left( t-0.5 \right) \right]{[1-F}_{H}\left( t \right)]$ (8)

## S2. Estimating the number of new HIV infections and the distribution of times from infection to diagnosis

The next step consisted in estimating the unknown parameters of the model (i.e. $\{ \lambda_{s}^{G_{1}}\}$, {$\lambda_{s}^{G_{2}}\}$, $\nu$ ,$\gamma$). As we split the population into two mutually exclusive groups (group 1 and group 2), the mean numbers of new HIV infections were estimated separately for each of the two groups.

Under our assumptions, the $Y_{t, D}$ were independent observations on Poisson variates, which gives the log-likelihood functions:

- for group 1: $\ln L\left( \lambda^{G_{1}} | {\{y}_{t, P}\} \right)\sim\sum_{t} \left( y_{t, P}\ln\mu_{t, P}-\mu_{t, P} \right)$ (9)
- for group 2: $\ln L\left( \nu, \gamma,\lambda^{G_{2}} | {\{y}_{t, H}\}, {\{y}_{t, A}\} \right)\sim\sum_{t} \left( y_{t, H}\ln\mu_{t, H}-\mu_{t, H}+y_{t, A}\ln\mu_{t, A}-\mu_{t, A} \right)$ (10)

where $\mu_{t, P}= E\left( Y_{t, P} \right)$ is given by (2), $\mu_{t, H}= E\left( Y_{t, H} \right)$ is given by (3) and $\mu_{t, A}= E\left( Y_{t, A} \right)$ is given by (4).

For group 1, maximum likelihood estimates of $\{ \tilde{\lambda}_{s}^{G_{1}}\}$were obtained by using the expectation-maximization-smoothing (EMS) algorithm [[3](#_ENREF_1)]. For group 2, following Becker *et al.* [[3](#_ENREF_1)], we derived estimates of the two unknown parameters of the distribution of the pre-AIDS HIV testing rate ($\tilde{\nu}$ ,$\tilde{\gamma}$) and the mean numbers of new HIV infections that are then diagnosed with AIDS or that are then diagnosed without AIDS or PHI ($\{ \tilde{\lambda}_{s}^{G_{2}}\}$) by using the Newton-Raphson method and the EMS algorithm (see ref. [[3](#_ENREF_1)] for more details). Finally, by adding together the estimates of the mean number of new infections in each group, ${\{\tilde{\lambda}}_{s}^{G_{1}}\}$ and $\{\tilde{\lambda}_{s}^{G_{2}}\}$, we obtained estimates of the mean numbers of new HIV infections ($\{\tilde{\lambda}_{s}\}$).

Using the group-specific estimates of the number of new HIV infections and the group-specific distributions of time from infection to diagnosis, we obtained the distribution of times from infection to diagnosis for individuals infected in quarter *s* as follows:

$\tilde{F}_{s}\left( t \right)=\frac{\sum_{x=1}^{t} \left( \tilde{\lambda}_{s}^{G_{1}}f_{x,P}+\tilde{\lambda}_{s}^{G_{2}}\left( \tilde{f}_{x,A}+\tilde{f}_{x,H} \right) \right)}{\tilde{\lambda}_{s}^{G_{1}}+\tilde{\lambda}_{s}^{G_{2}}}=\frac{\sum_{x=1}^{t} \left( \tilde{\lambda}_{s}^{G_{1}}f_{x,P}+\tilde{\lambda}_{s}^{G_{2}}\left( \tilde{f}_{x,A}+\tilde{f}_{x,H} \right) \right)}{\tilde{\lambda}_{s}}$ (11)

where $\tilde{\lambda}_{s}^{G_{1}}f_{x,P}+\tilde{\lambda}_{s}^{G_{2}}(\tilde{f}_{x,A}+\tilde{f}_{x,H})$ is the estimated number of individuals infected in quarter *s* who are diagnosed *x* quarters after contracting HIV, $\tilde{f}_{x,A}$ and $\tilde{f}_{x,H}$ are respectively the estimated probability that an infected individual is diagnosed with AIDS, *x* quarters after contracting HIV, and was not tested for HIV before developing AIDS and the estimated probability that an HIV-infected individual is diagnosed without AIDS or PHI, *x* quarters after contracting HIV, without first developing AIDS.

It is important to note that, although the group-specific distributions are stationary, the distribution $F_{s}\left( t \right)$ varies with time since $\tilde{\lambda}_{s}^{G_{1}}$ and $\tilde{\lambda}_{s}^{G_{2}}$ vary with time. Indeed, if individuals test for HIV more often, then more individuals are diagnosed in early stages of the HIV infection. Hence, the number of people diagnosed with PHI (i.e. group 1) increases, while the number of people diagnosed without PHI (i.e. group 2) decreases accordingly. As a result, the number of new infections among individuals belonging to group 1 ($\tilde{\lambda}_{s}^{G_{1}}$) increases, the number of new infections among individuals belonging to group 2 ($\tilde{\lambda}_{s}^{G_{2}}$) decreases, and the distribution of time from infection to diagnosis becomes shorter. Thus, our method allows for accounting some changes in test-seeking behaviors over calendar time.

## S3. Estimating the number of undiagnosed HIV-infected individuals

Using equation (11), we obtained the cumulative probabilities of not being diagnosed *t* quarters after contracting HIV according to the time of infection (*s*): $1-\tilde{F}_{s}\left( t \right)$. We then combined these cumulative probabilities with the estimated number of newly HIV-infected individuals at each point in time to estimate those who were still undiagnosed in quarter *t*:

$\tilde{U}_{t}=\sum_{s=1}^{t} \tilde{\lambda}_{s}(1-\tilde{F}_{s}\left( t \right))$ (12)

## S4. Precision of the estimates and estimates of the HIV incidence before 2003

We assessed the precision of the estimates by using a bootstrap procedure. New datasets were simulated by generating new realizations of $\left( Y_{t, P} \right), \left( Y_{t, H} \right)$ and $\left( Y_{t, A} \right)$ from the Poisson distributions with respective mean $\left( y_{t, P} \right), \left( y_{t, H} \right)$ and $\left( y_{t, A} \right)$. Using this procedure, we generated three thousand new datasets.

For each of the three thousand new datasets, we estimated the numbers of new HIV infections ($\{\tilde{\lambda}_{s}\}$) and the two parameters of the distribution of the pre-AIDS HIV testing rate ($\tilde{\nu}$ ,$\tilde{\gamma}$). As mentioned, in the main text, in France, case reporting of new HIV diagnoses was implemented from mid-2003. Therefore, we had observations for the number of new HIV diagnoses from 2004 to 2014. To fit the back-calculation model to the observed numbers of new diagnoses from 2004 to 2014, the annual numbers of new HIV infections before 2004 were assumed to be constant and were set *a priori*, while the numbers of new HIV infections from 2004 to 2014 were estimated as described in the section S2. The *a priori* values for the annual number of new HIV infections before 2004 were randomly drawn from a uniform distribution between 0 and 1.3 times the number of new diagnoses in 2004. Since we generated three thousand new datasets, three thousand *a priori* values were generated, and three thousand values of the numbers of new HIV infections from 2004 to 2014 and of the two parameters of the distribution of the pre-AIDS HIV testing rate were estimated. Then out of the three thousand estimated parameter sets, we selected the three hundred that maximized the likelihood given in equations (9) and (10). Note that our estimates of incidence, number of undiagnosed HIV infections and time to diagnosis in the recent past are not highly sensitive to our assumptions regarding values for the annual number of new HIV infections before 2004. From the three hundred selected parameter sets, we calculated mean estimates and 95% confidence intervals using the percentiles method, for the annual numbers of new HIV infections, from 2004 to 2014, the numbers of undiagnosed HIV infections in 2014 and the distributions of times from infection to diagnosis according the year of infection.

## S5. Estimating population sizes

To estimate population sizes at the national and subnational level, we used three data sources: The National Institute of Statistics and Economic Studies [[5](#_ENREF_1)] and two national surveys, one on sexual behavior [[6](#_ENREF_1)], and another one on drug use [[7](#_ENREF_1)].

We obtained data for the year 2014 from the National Institute of Statistics and Economic Studies, via the “Réseau Quetelet” [[8](#_ENREF_1)], on the size of adult populations aged 18-64 years by sex and country of birth at the national level and for each department of France. From these data, we calculated, for each sex, at the national and regional level, the size of adult populations aged 18-64 years born in France and abroad, and among those born abroad those born in sub-Saharan Africa (Table S1).

Men who have sex with men (MSM) were defined as men who had at least one sexual intercourse with another man in the past twelve months. We obtained estimates of the proportion of MSM, at the national and subnational level, from data collected in 2006 through the national survey on sexual behavior (Table S2). By multiplying these proportions to the corresponding size of adult men aged 18-64 years, we obtained estimates of the number of MSM at the national level and for each region of interest (Table S4).

People who inject drugs (PWID) were defined as individuals who injected drugs in the past twelve months. We obtained estimates of the number of men and women who injected drugs in the past twelve months, at the national and subnational level, from data collected in 2014 through national survey on drug use (Table S3). Separate estimates for men and women were available at the national level only; at the subnational level only overall estimates were available. We then used the national-level gender repartition (i.e. 76% of PWID were men and 24% were women) to estimate the number of male and female PWID in each region of interest.

We then estimated the number of heterosexual men and women, at the national and subnational level, by respectively subtracting from the men population the estimated numbers of MSM and male PWID and from the women population the estimated number of female PWID. We assumed that the prevalence of drug use and MSM was similar among individuals born in France and those born abroad.

Using mean and standard error of the estimated prevalence of drug use and MSM, we generated three hundred values for each population size and then obtained mean estimates (and 95% confidence intervals) of the numbers of MSM, PWID, heterosexuals, by sex and country of birth, at the national and subnational level (Table S4).

## S6. Estimating prevalence rates of undiagnosed HIV and rates of HIV incidence

To estimate the prevalence rates of undiagnosed HIV infections, for each group, we divided the estimated number of undiagnosed HIV-infected individuals by the corresponding population size estimates. Since, three hundred estimates of the number of undiagnosed HIV-infected individuals and of the corresponding population size were generated, we obtained three hundred estimates of the prevalence rates of undiagnosed HIV infections, from which we derived mean and 95% confidence intervals using the percentile method.

To estimate the rates of HIV incidence, for each group, we divided the estimated number of new HIV infections by the corresponding estimates of the number of individuals at risk of HIV infection. The number of individuals at risk of HIV infection was estimated for each group by subtracting to the estimated size of the group the estimated number of individuals living with HIV, using previously published group-specific prevalence rates of HIV in France [9] (Table S5); estimates of the HIV prevalence rates were only available at the national level, we then assumed similar HIV prevalence rates in the region of interest. Note that, except for MSM, subtracting the prevalence of HIV from the denominator only slightly changed the denominator, because the HIV prevalence was small. Regarding MSM, a recent HIV prevalence study conducted in France (not yet published) showed that HIV prevalence among MSM was high in many regions of France, hence it seemed also reasonable to assume that the prevalence of HIV among MSM in the region of interest was similar than the national-level HIV prevalence. Using mean and standard error of the estimated prevalence of HIV, we generated, for each group, three hundred values for the number of individuals living with HIV. Each of these three hundred values were randomly matched with one of the three hundred generated value for the size of the group to obtain three hundred values of the number of individuals at risk of HIV infection for each group. From the three hundred estimates of the number of new HIV infections and of the corresponding number of individuals at risk of HIV infection, we obtained three hundred estimates of the HIV incidence rates, from which we derived mean and 95% confidence intervals using the percentile method.

## S7. Statistical tests and maps

Statistical comparisons between groups, and between regions and national level, were carried out using Mann-Whitney test for incidence and undiagnosed prevalence rates, and using two-sided Kolmogorov-Smirnov test for the distribution of times between infection and diagnosis. Kruskal-Wallis test was used to test the statistical differences in national incidence each year from 2004 to 2014. Statistical analyses were performed using R3.2.4 [[1](#_ENREF_1)0] and maps were produced using the package maptools in R 3.2.4 [[1](#_ENREF_1)1].

## S8. Internal and external validity

We conducted some works to check the internal and external validity of our results.

**Internal validity**

First, all data were check for correctness in form and format. Second, we checked and compared trends for HIV incidence and HIV diagnosis. Specifically, it is important to check that the estimated numbers of newly infected are consistent with the observed numbers of newly diagnosed HIV cases. For example, it would not be consistent to have a much higher estimated numbers of new HIV infections than numbers of newly diagnosed HIV cases for years without having an increase in the numbers of newly diagnosed HIV cases at some point (except in the extreme scenario where some people decide not to be tested for HIV anymore). Other example, it would not be consistent to have a much lower estimated numbers of new HIV infections than numbers of newly diagnosed HIV cases for years without observing a decline in the numbers of newly diagnosed HIV cases at some point. We also checked whether, over the whole period, the estimated total number of new HIV infections is in the same order of magnitude than the observed total number of newly diagnosed HIV cases. Overall at the national level, there were 73897 newly diagnosed HIV cases between 2004 and 2014, and over the same period we estimated that 69039 (95% CI: 65679-73049) new HIV infections occurred. Third, we checked the adjustment of the model to ensure that the expected numbers of HIV diagnosis over 2004-2014 matched the observed numbers of HIV diagnosis. As an example, we show in Table S6 the expected and observed numbers of HIV diagnosis at the national level. It can be seen that overall our model fits pretty well the observed numbers of HIV diagnosis. We also projected forward the annual numbers of new HIV infections according to the annual distributions of time between infection and diagnosis to estimate the number of new HIV diagnosis that should be observed in 2015 at the national level. We obtained an estimate of 6408 (5848-7008) new HIV diagnoses for the year 2015, assuming that the incidence and the distribution of time from infection to diagnosis in 2015 remained equal to those estimates for 2014. Santé publique France, on the other hand, reported that 5997 (5806-6188) persons had been newly diagnosed with HIV in 2015 in France. Our prediction is thus compatible with what was reported for 2015; note that the small discrepancy could be due to our assumption that the incidence in 2015 remains equal to the one in 2014, especially as the observed number of new HIV diagnosis decreased from 2014 (6170) to 2015 (5997), so the HIV incidence could have decreased in 2015.

**External validity**

In our approach, we do not use data on CD4 count at HIV diagnosis, rather we use data on the clinical status at diagnosis. Thus, we used CD4 count at HIV diagnosis to broadly check the consistency of our results on the time from HIV infection to diagnosis in two ways. First, we checked whether our overall estimated time from HIV infection to diagnosis was consistent. With our approach, we estimated a median time from infection to diagnosis of 3.3 years. If we used data on CD4 count at diagnosis – in France, mean CD4 cell counts at diagnosis at the national level over the years 2011-2014 was 394 and median value was 370 – and “convert” this CD4 count into time since infection using estimates reported by the CASCADE collaboration -- Lodi et al. [[1](#_ENREF_1)2] estimated a median time from seroconversion to CD4 cell count <350 of 4.19 years (95% IC 4.09-4.28) – we can obtain that the time from infection to diagnosis should be lower than 4.2 years in France. This is consistent with our estimates. Note however that in our study, we estimate time from infection to diagnosis for individuals infected in a specific period, whereas when we used CD4 count at HIV diagnosis we obtain a broad idea of the time since infection for people diagnosed over a certain period, thus we do not compare exactly the same thing. Second, we checked whether the group-specific times from infection to diagnosis estimated by our model, and especially their ranking, were consistent with observed group-specific data on CD4 cell count at HIV diagnosis (see Table S7). We found that MSM and heterosexual women born in France had equivalent CD4 cell count at diagnosis and higher CD4 cell count than heterosexual men, whether born in France or abroad, and PWID. This is consistent with our estimated median time from infection to diagnosis (Table S7). Born-abroad heterosexual women had lower CD4 cell count at diagnosis than heterosexual women born in France, whereas we estimated similar median times from infection to diagnosis for these two groups. This apparent inconsistency can however be explained when considering the fact that most born-abroad heterosexual women are women born in sub-Saharan Africa. Indeed, it has been shown that CD4 cell count at seroconversion and CD4 decline are different for individuals born in SSA than for individuals born in Europe [[1](#_ENREF_1)3], and this explain why born-abroad heterosexual women can have similar estimated time from HIV infection to diagnosis than that estimated for women born in France while having lower CD4 count at HIV diagnosis; similar phenomenon can explain the differences observed between born-abroad heterosexual men and heterosexual men born in France.

Third, our estimated time from HIV infection to diagnosis for MSM in France (median of 2.7 years (0.5-5.0)) was similar to the one estimated for MSM in the Netherlands (average of 2.6 years (2.3-3.0)) [[1](#_ENREF_1)4] and to that estimated for MSM in UK (average of 3.2 years (2.6-3.8)) [[1](#_ENREF_1)5]

Finally, we compared our estimates for the number of undiagnosed HIV infections with another estimate. In our study, we estimated that 24197 (22296-25944) persons aged 18-64 were living with undiagnosed HIV in 2014 in France. Cazein et al. [[1](#_ENREF_1)6] found that 29,008 persons aged 18–80 years were living with undiagnosed HIV in mainland France (95% confidence interval: 11,603 to 70,958) in 2004. As aforementioned, according to our study, over the 2004-2014 period there were slightly more people diagnosed with HIV (73897 cases) than people newly infected with HIV (69039 new HIV infections); there is a difference of 4858 cases. If we subtract this difference to the number of people living with undiagnosed HIV estimated in 2004 by Cazein et al. [[1](#_ENREF_1)6] we obtained that in 2014 there were 24150 people living with undiagnosed HIV. This is in very good agreement with our estimate of the number of undiagnosed infections for the year 2014.

**References**

1. Ndawinz JD, Costagliola D, Supervie V. New method for estimating HIV incidence and time from infection to diagnosis using HIV surveillance data: results for France. AIDS. 2011;25(15):1905-13.

2. Supervie V, Ndawinz JD, Lodi S, Costagliola D. The undiagnosed HIV epidemic in France and its implications for HIV screening strategies. AIDS. 2014;28(12):1797-804.

3. Becker NG, Lewis JJ, Li Z, McDonald A. Age-specific back-projection of HIV diagnosis data. Stat Med 2003;22:2177-90.

4. Brookmeyer R, Goedert JJ. Censoring in an epidemic with an application to hemophilia-associated AIDS. Biometrics. 1989;45:325335.

5. National Institute of Statistics and Economic Studies (INSEE). <https://www.insee.fr/en/accueil>

6. Bajos N, Bozon M. Enquête sur la sexualité en France : pratiques, genre et santé: Paris: Editions La Découverte; 2008.

7. Janssen E. Estimating the number of people who inject drugs: A proposal to provide figures nationwide and its application to France Journal of Public Health. 2017;Accepted.

8. Données Harmonisées des recensements de la population : Tabulation sur mesure, INSEE [producteur], ADISP-CMH [diffuseur].

9. Supervie V, Marty L, Lacombe JM, Dray-Spira R, Costagliola D, FHDH-ANRS CO4 study group. Looking Beyond the Cascade of HIV Care to End the AIDS Epidemic: Estimation of the Time Interval From HIV Infection to Viral Suppression. J Acquir Immune Defic Syndr. 2016;73(3):348-55.

10. R Core Team (2016). R: A language and environment for statistical computing. R Foundation for Statistical Computing, Vienna, Austria. URL https://www.R-project.org/.

11. Bivand R and Lewin-Koh N (2016). maptools: Tools for Reading and Handling Spatial Objects. R package version 0.8-39. <https://CRAN.R-project.org/package=maptools>.

12. Lodi S, Phillips A, Touloumi G et al. Time from human immunodeficiency virus seroconversion to reaching CD4+ cell count thresholds <200, <350, and <500 Cells/mm³: assessment of need following changes in treatment guidelines. Clin Infect Dis. 2011 ; 53(8):817-25.

13. Pantazis N, Morrison C, Amornkul PN et al. Differences in HIV natural history among African and non-African seroconverters in Europe and seroconverters in sub-Saharan Africa. PLoS One. 2012;7(3):e32369.

14. van Sighem A, Nakagawa F, De Angelis D et al. Estimating HIV Incidence, Time to Diagnosis, and the Undiagnosed HIV Epidemic Using Routine Surveillance Data. Epidemiology. 2015;26(5):653-60

15. Birrell PJ^1^, Gill ON, Delpech VC et al. HIV incidence in men who have sex with men in England and Wales 2001-10: a nationwide population study. Lancet Infect Dis. 2013; 13(4):313-8.

16. Cazein F, Barin F, Le Strat Y et al. Prevalence and characteristics of individuals with undiagnosed HIV infection in France: evidence from a survey on hepatitis B and C seroprevalence. J Acquir Immune Defic Syndr. 2012 Aug 1;60(4):e114-7

**Tables**

**Table S1: Population size, aged 18-64, by sex and origin, in 2014 in France at the national level and in four selected regions [5,8]**

|  | France | Ile-de-France | Provence-Alpes-  Côte d’Azur | Centre | Guyane |
| --- | --- | --- | --- | --- | --- |
| Men | 19410383 | 3676384 | 1411695 | 738717 | 69020 |
| Men born in France | 16748843 | 2682568 | 1153431 | 664183 | 38447 |
| Born-abroad men | 2661540 | 993817 | 258264 | 74534 | 30573 |
| Men born in SSA | 468671 | 234855 | 30859 | 15301 | 635 |
| Women | 19998673 | 3882308 | 1491138 | 753568 | 71581 |
| Women born in France | 17133265 | 2799623 | 1210125 | 675481 | 38787 |
| Born-abroad women | 2865407 | 1082685 | 281013 | 78087 | 32794 |
| Women born in SSA | 417221 | 212487 | 26234 | 13153 | 771 |
| Total born in France | 33882109 | 5482191 | 2363556 | 1339664 | 77234 |
| Total born-abroad | 5526947 | 2076501 | 539277 | 152621 | 63367 |
| Total | 39409056 | 7558693 | 2902833 | 1492285 | 140601 |

SSA: Sub-Saharan Africa

**Table S2: Estimated proportion of men, aged 18-69, who had sex with another man in the past twelve months in France and in four selected regions in 2006 [**[**6**](#_ENREF_1)**]**

|  | France | Ile-de-France | Provence-Alpes-  Côte d’Azur | Centre | Guyane |
| --- | --- | --- | --- | --- | --- |
| Proportion of men who had sex with another man in the past 12 months (95 % CI) | 1.6  (1.3-2.0) | 2.5  (1.6-3.8) | 3.0  (1.7-5.4) | 0.9  (0.3-2.5) | xx |

xx: Estimate was not available for Guyane because this region was not surveyed; CI: Confidence interval.

**Table S3: Estimated number of individuals, aged 15-64, who injected drugs in the past twelve months, by sex, in France and in four selected regions in 2014 [7]**

|  | France | Ile-de-France | Provence-Alpes-  Côte d’Azur | Centre | Guyane |
| --- | --- | --- | --- | --- | --- |
| Number of male PWID (95 % CI) | 80000  (61200-106200) | * | * | * | xx |
| Number of male PWID (95 % CI) | 25000  (16100-38900) | * | * | * | xx |
| Total number of PWID (95 % CI) | 105000  (85300-130000) | 25900  (20200-35800) | 15500  (9700-27900) | 2400  (1300-6000) | xx |

PWID: persons who inject drugs; CI: confidence interval; xx: Estimates were not available for Guyane because this region was not surveyed; *the survey provided separate estimates for men and women at the national level only; at the subnational level only overall estimates were available.

**Table S4: Estimated population sizes and 95% confidence intervals, aged 18-64, by sex, HIV exposure group and origin, in France and in four selected regions**

|  | France | Ile-de-France | Provence-Alpes-  Côte d’Azur | Centre | Guyane |
| --- | --- | --- | --- | --- | --- |
| MSM (all) | 311480  (248800-382887) | 93178  (61341-134306) | 43515  (24506-70525) | 7011  (3211-13195) | xx |
| MSM born in France | 268770  (214684-330385) | 67989  (44759-98000) | 35554  (20023-57623) | 6304  (2887-11863) | xx |
| Born-abroad MSM | 42710  (34115-52501) | 25188  (16582-36306) | 7961  (4483-12902) | 707  (324-1331) | xx |
| Heterosexual women born in France | 17111885  (17100164-17119422) | 2795141  (2794084-2796114) | 1207109  (1205637-1208222) | 674966  (674599-675200) | 38787 |
| Born-abroad heterosexual women | 2861832  (2859872-2863092) | 1080951  (1080543-1081327) | 280313  (279971-280571) | 78027  (77985-78054) | 32794 |
| Heterosexual women  born in SSA | 416700  (416414-416883) | 212146  (212066-212220) | 26169  (26137-26193) | 13143  (13136-13147) | 770 |
| Heterosexual men born in France | 16411594  (16344929-16469836) | 2600331  (2568873-2623902) | 1108378  (1084438-1124669) | 656254  (650563-659892) | 38447 |
| Born-abroad heterosexual men | 2607948  (2597354-2617203) | 963350  (951696-972082) | 248176  (242816-251824) | 73644  (73006-74053) | 30573 |
| Heterosexual men  born in SSA | 459234  (457369-460864) | 227655  (224901-229719) | 29654  (29013-30090) | 15118  (14987-15202) | 635 |
| PWID (all) | 104317  (80727-132800) | 25742  (20723-30602) | 15343  (10229-21242) | 2382  (1410-3581) | xx |
| PWID born in  France | 89860  (69541-114382) | X | X | X | xx |
| PWID born abroad | 14458  (11185-18418) | X | X | X | xx |
| Total Men | 19410383 | 3676384 | 1411695 | 738717 | 69020 |
| Total Women | 19998673 | 3882308 | 1491138 | 753568 | 71581 |
| Total | 39409056 | 7558692 | 2902833 | 1492285 | 1406001 |

xx: Estimates were not available for Guyane because this region was not surveyed; MSM: men who have sex with men; PWID: persons who inject drugs; SSA: Sub-Saharan Africa. X Since, for PWID, estimates of HIV incidence, time to diagnosis and undiagnosed prevalence could not be produced according to the country of birth at the subnational level due to insufficient HIV cases, we did not estimate the number of PWID according to the country of birth at the subnational level.

**Table S5: Estimated national-level rates of HIV prevalence in France in 2010 by sex, HIV exposure group and origin**

|  | HIV prevalence rates (95% CI) |
| --- | --- |
| MSM | 17.00  (16.42-17.90) |
| Heterosexual women born in France | 0.12  (0.10-0.13) |
| Born-abroad heterosexual women | 1.58  (1.45-1.76) |
| Heterosexual men born in France | 0.12  (0.10-0.14) |
| Born-abroad heterosexual men | 1.07  (0.88-1.26) |
| PWID | 17.04  (15.93-19.01) |
| Total Men | 0.51  (0.49-0.54) |
| Total Women | 0.25  (0.23-0.26) |
| Total | 0.38  (0.36-0.39) |

MSM: men who have sex with men; PWID: persons who inject drugs; CI: confidence interval.

**Table S6: Annual expected and observed numbers of HIV diagnosis at the national level over 2004-2014**

|  | **Expected number of HIV diagnosis**  **(95% CI)** | **Observed number of HIV diagnosis**  **(95% CI)** |
| --- | --- | --- |
| **2004** | 7510  (5390-9292) | 7935  (7541-8329) |
| **2005** | 7504  (5790-8880) | 7649  (7327-7971) |
| **2006** | 7356  (6142-8267) | 7250  (6905-7595) |
| **2007** | 7059  (6342-7574) | 6675  (6355-6995) |
| **2008** | 6703  (6381-6961) | 6460  (6253-6667) |
| **2009** | 6437  (6268-6539) | 6372  (6122-6622) |
| **2010** | 6289  (6150-6435) | 6398  (6135-6661) |
| **2011** | 6194  (6050-6390) | 6292  (6079-6505) |
| **2012** | 6236  (6100-6404) | 6371  (6184-6558) |
| **2013** | 6263  (6140-6400) | 6325  (6029-6621) |
| **2014** | 6358  (6198-6532) | 6170  (5869-6471) |

CI: confidence interval.

**Table S7: Estimated time from infection to HIV diagnosis and CD4 cell count at HIV diagnosis by group**

|  | **Median time (in years) from infection to diagnosis (IQR)** | **Median CD4 cell count at diagnosis (IQR)** |
| --- | --- | --- |
| **MSM (all)** | 2.7  (0.5-5.0) | 433  (266-604) |
| **MSM born in France** | 2.6  (0.5-5.0) | 445  (274-616) |
| **Born-abroad MSM** | 3.2  (1.2-5.5) | 384  (222-545) |
| **Born-abroad heterosexual women (all)** | 3.1  (1.3-5.1) | 324  (171-504) |
| **Heterosexual women born in SSA** | 2.9  (1.2-4.9) | 320  (173-499) |
| **Born-abroad heterosexual men (all)** | 4.5  (2.4-6.8) | 271  (100-450) |
| **Heterosexual men born in SSA** | 4.2  (2.1-6.4) | 283  (116-455) |
| **Heterosexual women born in France** | 3.0  (1.0-5.4) | 414  (210-608) |
| **Heterosexual men born in France** | 4.6  (1.9-7.0) | 315  (117-505) |
| **PWID (all)** | 4.0  (1.8-6.5) | 300  (130-505) |
| **PWID born in France** | 4.3  (2.1-6.7) | 311  (118-503) |
| **PWID born abroad** | 3.5  (1.5-6.0) | 293  (143-520) |
| **Total men** | 3.4  (1.1-5.8) | 381  (193-560) |
| **Total women** | 3.1  (1.2-5.0) | 348  (178-535) |
| **Total** | 3.3  (1.2-5.7) | 370  (88-553) |

MSM: men who have sex with men; PWID: persons who inject drugs; SSA: Sub-Saharan Africa; CI: confidence interval. IQR: inter-quartile range.

**Figure Legends**

**Figure S1: Surveillance data on newly diagnosed HIV cases.** (A) Annual national number of newly diagnosed HIV cases and 95% confidence intervals from 2004 to 2014; (B) Average annual number of new HIV diagnoses over the years 2009-2014 in each French region and percentage of new HIV diagnoses by HIV exposure group and origin. Overall, 10% of newly diagnosed HIV cases were diagnosed during the primary HIV infection (PHI), 15% with AIDS, and 75% without AIDS or PHI.

**Figure S1**


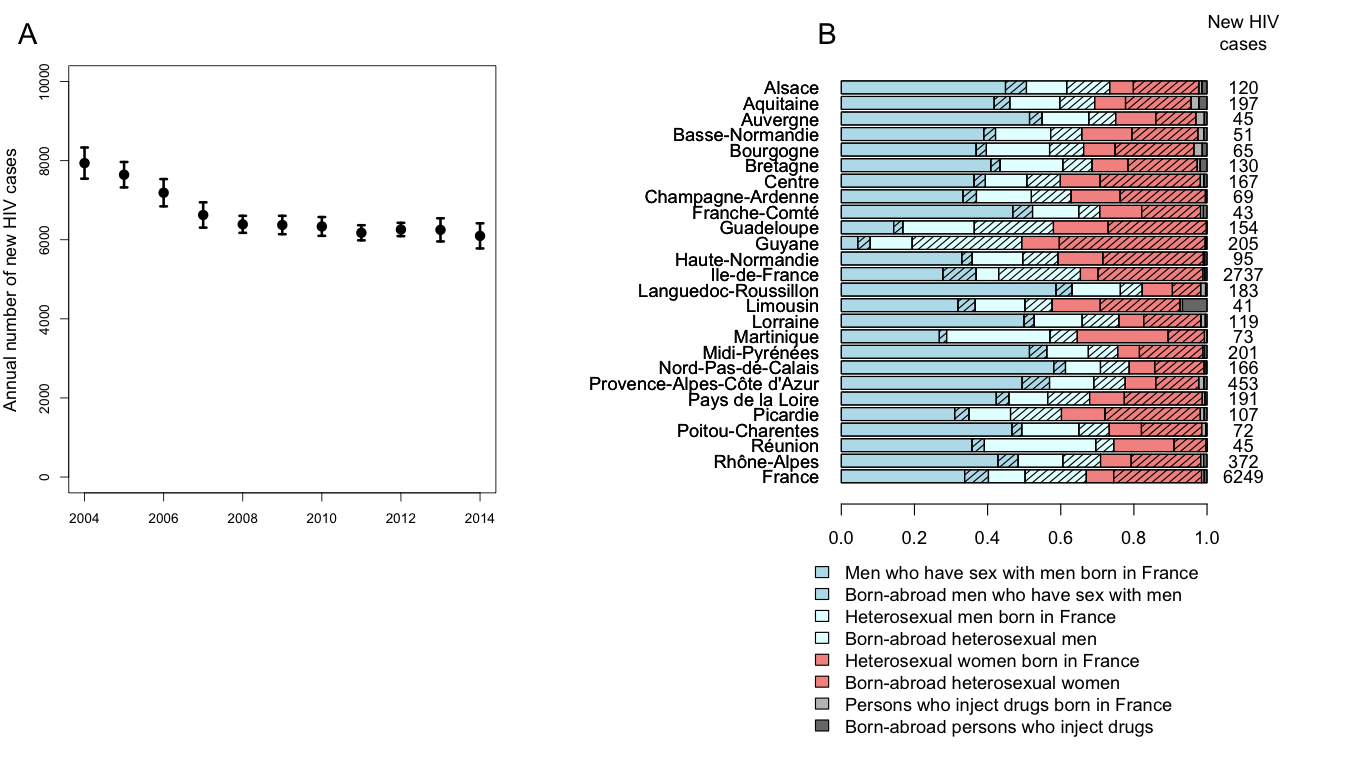

Supplement: Supplementary file 1 — Appendix S1. Description of the back‐calculation model. Appendix S2. Estimating the number of new HIV infections and the distribution of times from infection to diagnosis. Appendix S3. Estimating the number of undiagnosed HIV‐infected individuals. Appendix S4. Precision of the estimates and estimates of the HIV incidence before 2003. Appendix S5. Estimating population sizes. Appendix S6. Estimating prevalence rates of undiagnosed HIV and rates of HIV incidence. Appendix S7. Statistical tests and maps. Appendix S8. Internal and external validity. Table S1. Population size, aged 18 to 64, by sex and origin, in 2014 in France at the national level and in four selected regions 5, 8 Table S2. Estimated proportion of men, aged 18 to 69, who had sex with another man in the past twelve months in France and in four selected regions in 2006 6 Table S3. Estimated number of individuals, aged 15 to 64, who injected drugs in the past twelve months, by sex, in France and in four selected regions in 2014 7 Table S4. Estimated population sizes and 95% confidence intervals, aged 18 to 64, by sex, HIV exposure group and origin, in France and in four selected regions Table S5. Estimated national‐level rates of HIV prevalence in France in 2010 by sex, HIV exposure group and origin Table S6. Annual expected and observed numbers of HIV diagnosis at the national level over 2004 to 2014 Table S7. Estimated time from infection to HIV diagnosis and CD4 cell count at HIV diagnosis by group Figure S1. Surveillance data on newly diagnosed HIV cases. (A) Annual national number of newly diagnosed HIV cases and 95% confidence intervals from 2004 to 2014; (B) average annual number of new HIV diagnoses over the years 2009 to 2014 in each French region and percentage of new HIV diagnoses by HIV exposure group and origin. Overall, 10% of newly diagnosed HIV cases were diagnosed during the primary HIV infection (PHI), 15% with AIDS and 75% without AIDS or PHI. [file JIA2-21-e25100-s001.docx]
